# Commencement Exercises

**Published:** 1902-05

**Authors:** 


### COMMENCEMENT EXERCISES.

### KANSAS CITY VETERINARY COLLEGE.

The eleventh annual commencement exercises of this college were held in the college hall, March 12th, 7.30 P.M. The Faculty address was delivered by Hon. J. A. McLane. Diplomas were presented by Dr. R. C. Moore to the following: James B. Boazman, James M. Lawrence, James Mahon, Henry R. McNally, E. Manford Nighbert, Robert A. Phillips, Erni V. Robnett, Morey A. Sappington, Frank M. Starr, Orville A. Stingley, Fred W. Weston, and Zachary Veldhius, D.V.S. (post-graduate). Class response was by Dr. James Mahon. The hall was filled to overflowing with the friends of the students, and the programme proved delightful to all.

On the 6th ult. the college gave its annual dinner to the students, Faculty, and friends of the college, at the Coates House, with about 140 covers. The *menu* was served in a most pleasing manner, Dr. R. C. Moore, acting as toast-master. The following toasts were responded to: "Ethics," Hon. J. A. McLane; "Three Years," Fred. W. Weston; "The Middle of the Way," Lloyd Champlain; "Freshmen," Chas. E. Wiggins; "K. C. V. C. Alumni," Dr. B. F. Kaupp; "The Pathogenic Germ," Dr. I. J. Wolf; "The Bureau of Animal Industry," Dr. A. G. G. Richardson; "The Veterinary Profession from a Layman's Point of View," Hon. Alfred Weston; "The K. C. V. C.," Dr. S. Stewart.

---

### THE WESTERN VETERINARY COLLEGE.

The fifth regular session of this college closed on March 19, 1902, with nineteen graduates, who received their diplomas. Following are their names and addresses: Charles W. Bigarel, Wendell, Minn.; Judson R. Flanders, Cameron, Mo.; Roy Grimsley, Americus, Kas.; W. H. Gatchell, Kansas City, Mo.; Fred G. Cook, Carthage, Mo.; W. Harry Lynch, Beverly, Mass.; James R. Petty, Winston, N. C.; Harvey E. McGee, Osceola, Iowa; Howard G. Merwin, Dowagiac, Mich.; A. A. Motley, Alpena, Mich.; Urie B. Reynolds. Cynthiana, Ind.; Timothy Riley, Canton, Ill.; James H. Scott, Newton, Iowa; John Servatius, Ottawa, Kas.; William

J. Wilson, Canton, Ill.; George E. Wallace, Tracy, Minn.; Charles Weeks, Logan, Iowa; Joseph Wenger, Dutton, Mich.; E. C. Whitaker, Oak Ridge, Mo.

---

### McGILL UNIVERSITY.

THE annual convocation for the conferring of degrees in comparative medicine and veterinary sciences at McGill University was held March 27, 1902. Dr. Craik presided; Sir William McDonald represented the governors, and Principal Peterson was there as vice-chancellor. Others present were: Dr. Duncan McEachran, dean of the Faculty; Dean Johnson, Dean Bovey, Dean Roddick, Dean Walton, Mr. Justice Davidson, Dr. Charles McEachran, Dr. Wesley Mills, Dr. Morrow, Dr. Ruttan, Dr. Girdwood, Professor Penhallow, Professor Capper, Professor Chandler, Mr. W. Vaughan, Mr. W. A. Nicholson, Mr. Recorder Weir, and Mr. Chas. Alexander.

Dr. A. R. Douglas read the class valedictory, in which he dwelt on their gratitude for the zealous and conspicuously able instruction of the professors, and urged the difficulties, responsibilities, and importance of the veterinary calling.

Dr. McEachran wished the students God-speed, saying no school in the Dominion had turned out so many leading veterinarians as the McGill Faculty, hampered in many ways as it is. He urged the great importance of the science. When individual horses are valued at $100,000, and cows at $15,000, and dogs at $10,000, the need of trained veterinary doctors is apparent. In the United States there are $2,000,000,000 invested in stock, and an annual loss of $20,000,000 from hog cholera alone. Moreover, the welfare of agriculture and the public food supply depend largely upon them. He went on to deal with the growing recognition of the sciences in the American colleges, and pointed out the necessity of endowments for the carrying on of what is only one branch of the great science of medicine. The Provincial Legislature had at last recognized them by passing an act to regulate the right to assume the title and to practise.

Dr. Peterson expressed his sympathy with the work of the Faculty, saying that at the University of Pennsylvania the efficiency of the veterinary faculty is continually rising, and he was extremely pleased at the decision of Harvard to take up their work in comparative medicine again with some of the endowments lately given for the study of medicine. He concluded with a few words of commendation of the work done by the students and professors.

Dr. Craik said it had been one of his first duties as dean of the Faculty of Medicine to assist in the incorporation of the veterinary school in the university. He would remind them that his own Faculty had struggled along for years before reaching its present position of stability, and he believed their sun of prosperity would soon rise.

The candidates for the degree of D. V. S. were capped by Principal Peterson, as follows: W. Reid Blair, Alexander R. Douglas, Seymour Hadwen, A. D. Harrington, G. A. Kennedy, W. Manchester (in absentia), W. H. Spear, and J. W. Symes.

Medal for best examinations during the three-years' course, A. D. Harrington; prizes for veterinary medicine and surgery, A. R. Douglas; cattle pathology, A. D. Harrington; materia medica, A. D. Harrington; anatomy, T. C. Hays.

Extra prizes for the best essay read before the Veterinary Medical Association: 1st, A. D. Harrington; 2d, A. R. Douglas; 3d, W. R. Blair. For the best essay read before the Psychological Society: 1st, A. D. Harrington; 2d, F. M. Gray.

---

### McKILLIP VETERINARY COLLEGE.

A NEW era was inaugurated at the sixth annual commencement exercises of this college. The growth of the institution rendered it necessary to obtain a larger hall than in former years to accommodate the guests; in consequence the exercises were held at the auditorium of the Y. M. C. A. building, Chicago, Ill., March 28th, at 7.30 P.M. The exercises were opened by the Rev. Pleasant Hunter, D.D., after which followed the conferring of the degree of the college (M.D.V.) by Dr. M. H. McKillip upon the following gentlemen: C. P. Draper, A. H. Fehr, Robert Frame, Charles Frazier, T. P. Galbraith, H. L. Jackson, George Jerome, John Keppel, C. A. Mack, S. H. Miller, C. W. Moore, M. W. Shempf, J. F. Sylvester, B. C. Tillman, Thomas Trinder, F. R. Whipple, T. T. Kendrew, J. P. Luxmore, H. A. Walker.

The address of the evening was delivered by Dr James G. Kiernan, and prizes were awarded by the Secretary, Dr. John J. Millar, as follows: Highest average for three years, John Keppel (presented by the McKillip Veterinary College); highest average for senior year, John Keppel (presented by Faculty of McKillip Veterinary College); highest average for junior year, W. G. Langley (presented by Faculty); highest average for freshman year, E. D. Anderson (presented by Faculty); highest average in bacteriology, John Keppel (presented by Prof. I. D. Rawlings); highest average in anatomy (freshman), A. Paul (presented by Prof. F. S. Schoenleber); highest average in materia medica, M. W. Schultz (presented by Prof. T. B. Newby).

Following the exercises about 150 persons adjourned to the banqueting hall, where a most elaborate menu was given in honor of the class of 1902. Toast-master Prof. E. M. Reading contributed greatly to the success of the evening's entertainment; Howard L. Jackson responded for the graduating class; W. G. Langley of the senior class, and J. W. Eastland of the junior class. The different members of the Faculty, as they were respectively called upon by the toastmaster, responded, and in many instances were very witty and amusing. Rev. Pleasant Hunter paid a very high tribute to the graduates, and gave to them a talisman for their future guidance. A very regretable feature was the absence from the banqueting table of Dr. McKillip, who, on account of his recent severe illness, deemed it advisable not to remain after the graduating exercises.

An innovation in the history of the McKillip Veterinary College was marked by the final exercises of the class of 1902.

---

### GRAND RAPIDS VETERINARY COLLEGE.

THE fifth annual commencement exercises of this college were held on the evening of March 28th at 215 Butterworth Avenue, Grand Rapids, Mich. Dr. J. B. Griswold, President of the college, gave the opening address, and told of the many failures and triumphs of the members of the class of 1902. He also gave them valuable advice. The Rev. R. H. Fortescue Gairdner delivered an address. He spoke of the higher attainments of the profession, asking the graduates to not only make successful records as professional men, but to always carry manliness with their medicine cases. Professor Bessey gave a scientific address, dealing with the history of the science from a physiological stand-point. Fred. L. Small, the valedictorian, delivered an address on the future of the individual members of the class. This was followed by remarks of Dr. L. L. Conkey, founder of the school, and Dr. William A. McLean, Dean. Judge Willis B. Perkins, President of the Board of Trustees of the college, made a few remarks and presented the diplomas to the twenty-three graduates: A. Beck, Auburn, Iowa; F. Brouwer, Holland, Mich.; E. Boesewetter, West Bend, Wis.; E. Branyan, Bronson, Mich.; H. J. Getman, Traverse City, Mich.; George Rainy Gaggin, Australia; C. E. Greenwalt, Topeka, Ind.; James F. Hanley, Boston, Mass.; W. A. Haynes, Jackson, Mich.; A. G. Hersey, Grand Rapids, Mich.; E. L. Krieger, Benton Harbor, Mich.; F. S. Kinison, Dawson, Pa.; W. G. V. Lyons, South Norwalk, Conn.; Elmer D. Nash, Helena, Mont.; M. L. Pattison, Ridgeway, Mich.; Herman F. Sass, Toledo, Ohio; E. J. Sowerby, Rockford, Mich.; Fred. L. Small, Beulah, Mich.; J. F. Sudman, Boyne City, Mich.; A. R. Trickel, Brownton, Wis.; Harry W. Wise, Rife, Pa.; Joseph Wardle, Flint, Mich.; W. W. Sammit, Indianapolis, Ind.

Dr. W. A. McLean, of Greenville, Mich., took a post-graduating course; Dr. W. E. Beesey, of Grand Rapids, Mich., and Dr. J. E. Jaynes, of De Witt, Mich., received the honorary degree of Doctor of Veterinary Science.

---

Two Successful Cases of Arytenectomy. Detante operated upon two cases. The first was a Belgian horse which roared for four and one-half years. The operation was performed in the usual manner in 1900, and in eight weeks the roaring had disappeared. The horse has not roared up to the present.

The second case was a half-bred operated on in May, and in August did not roar at his highest speed.—*Rec. de Med. Vet.*

Trichloride of Iodine in Distemper. Zimmerman, of the Budapest Clinic, has experimented with trichloride of iodine in distemper in different breeds of dogs, 5 c.c. of 1 to 2000 aqueous solution, given hypodermically. From numerous observations the author concludes that this agent has given especially good results at the beginning of the disease. It was most efficacious in the gastro-intestinal form. Its prophylactic properties have not been tested.—*Osterreich Monats.*

Drs. Carter and Conard, of Philadelphia, conducted a newspaper controversy in March relative to the conditions of cleanliness, etc., that applies to the milk supply of the "City of Brotherly Love." All of their allusions to the two sides of this question were not of the most brotherly kind.

